# Nab-paclitaxel and gemcitabine plus camrelizumab and radiotherapy versus nab-paclitaxel and gemcitabine alone for locally advanced pancreatic adenocarcinoma: a prospective cohort study

**DOI:** 10.1186/s13045-023-01422-8

**Published:** 2023-03-20

**Authors:** Shuling Chen, Jiaxin Li, Aoran Dong, Zelong Liu, Meiyan Zhu, Meng Jin, Guangyan Wei, Shuang Wu, Yan Wang, Yong Chen, Zhenwei Peng

**Affiliations:** 1 Institute of Diagnostic and Interventional Ultrasound, Clinical Trials Unit, The First Affiliated Hospital, Sun Yat-Sen University, Guangzhou, 510080 China; 2 Department of Radiation Oncology, The First Affiliated Hospital, Sun Yat-Sen University, Guangzhou, 510080 China; 3 Institute of Precision Medicine, The First Affiliated Hospital, Sun Yat-Sen University, Guangzhou, 510080 China; 4 Cancer Center, The First Affiliated Hospital, Sun Yat-Sen University, Guangzhou, 510080 China

**Keywords:** Radiotherapy, Immunotherapy, Chemotherapy, Locally advanced pancreatic adenocarcinoma

## Abstract

Treatment options specifically for patients with locally advanced pancreatic adenocarcinoma (LAPC) are scare and chemotherapy alone delivers limited efficacy. Immunotherapy and radiotherapy are potential effective treatments for LAPC, and both of them may synergize with chemotherapy. Therefore, in this prospective cohort study, we compared the efficacy and safety of nab-paclitaxel plus gemcitabine combined with anti-programmed cell death (PD-1) immunotherapy and radiotherapy (hereafter, combination treatment) versus nab-paclitaxel plus gemcitabine (chemotherapy alone) in the treatment of LAPC. In the combination group, participants received conventional fractionated radiotherapy with doses ranging from 54 to 63 Gy in 28 fractions, intravenous camrelizumab 200 mg once every 3 weeks, and intravenous nab-paclitaxel plus gemcitabine on day 1 and 8 of a 21-day cycle for eight cycles until disease progression, death or unacceptable toxicity. In the chemotherapy group, participants received intravenous nab-paclitaxel plus gemcitabine on day 1 and 8 of a 21-day cycle for eight cycles. From April, 2020 to December, 2021, 96 participants with LAPC were prospectively enrolled with 32 received combination treatment and 64 received chemotherapy alone at a single center. The combination treatment yielded significantly longer median overall-survival (22.3 months vs. 18.6 months, *P* = 0.031) and progression-free survival (12.0 months vs. 10.5 months, *P* = 0.043) than chemotherapy alone did. And the incidence of severe adverse events was not significantly different between the combination group and chemotherapy group (*P* = 0.856). In conclusion, nab-paclitaxel plus gemcitabine combined with anti-PD-1 immunotherapy and radiotherapy was effective and safe for LAPC patients, and it warrants further investigation in larger randomized trials.

**Supplementary Information:**

The online version contains supplementary material available at 10.1186/s13045-023-01422-8.

**To the editor,**

Locally advanced pancreatic adenocarcinoma (LAPC) accounts for a sizeable proportion of pancreatic cancer, which is one of the most lethal cancers globally among all cancers [1]. However, the optimal management of LAPC remains an open question, due to the dismal therapeutic efficacy and scarce of prospective treatment data specifically in LAPC.

Chemotherapy alone (i.e., gemcitabine plus albumin-bound paclitaxel) delivers limited efficacy for LAPC [2–4]. For LAPC in which microscopic metastatic disease was present, novel therapies that can enhance local control while having systematic efficacy to control microscopic metastatic lesions may have the greatest potential for LAPC [5]. Chemoradiation could deliver a systematic benefit during local tumor control to reduce the opportunity of occult progression of pancreatic cancer [6, 7]. On the other hand, anti-programmed cell death-1 (PD-1) immunotherapy can synergize with chemotherapy to reduce tumor burden by alleviating chemotherapy resistance and modifying microenvironment [8]. Besides, it was also reported to synergize with radiotherapy by promoting T-cell priming with immunogenic cell death and reversing immunosuppressive microenvironment [9–12]. Thus, there is a rationale to combine these three treatments to promote both of the local and systematic tumor control. However, there is a lack of clinical data in this aspect.

Therefore, we performed this prospective cohort study to compare the efficacy and safety of nab-paclitaxel plus gemcitabine combined with anti- PD-1 immunotherapy and radiotherapy (combination treatment) versus nab-paclitaxel plus gemcitabine (chemotherapy alone) for LAPC patients. We enrolled treatment-naïve, histologically or cytologically confirmed LAPC patients who received one of these two treatments according to the inclusion and exclusion criteria (Additional file 1: Methods). In the combination group, participants received conventional fractionated radiotherapy with doses ranging from 54 to 63 Gy in 28 fractions, intravenous camrelizumab 200 mg once every 3 weeks, and intravenous nab-paclitaxel plus gemcitabine on day 1 and 8 of a 21-day cycle for eight cycles until disease progression, death or unacceptable toxicity (Fig. 1a). In the chemotherapy group, participants received intravenous nab-paclitaxel plus gemcitabine on day 1 and 8 of a 21-day cycle for eight cycles (Fig. 1a). All patients were informed of the advantages and disadvantages of the two treatment options, including potential treatment outcomes, treatment-related morbidities and costs, and the final treatment decision was generally made by the patients. Other detailed methods are described in Additional file 1: Methods. From April, 2020 and December, 2021, 96 participants were finally enrolled with 32 received combination treatment and 64 received chemotherapy alone (Additional file 2: Fig. S1). There was no significant difference in any baseline characteristics between these two groups (all *P* > 0.05, Additional file 3: Table S1).

**Fig. 1:**
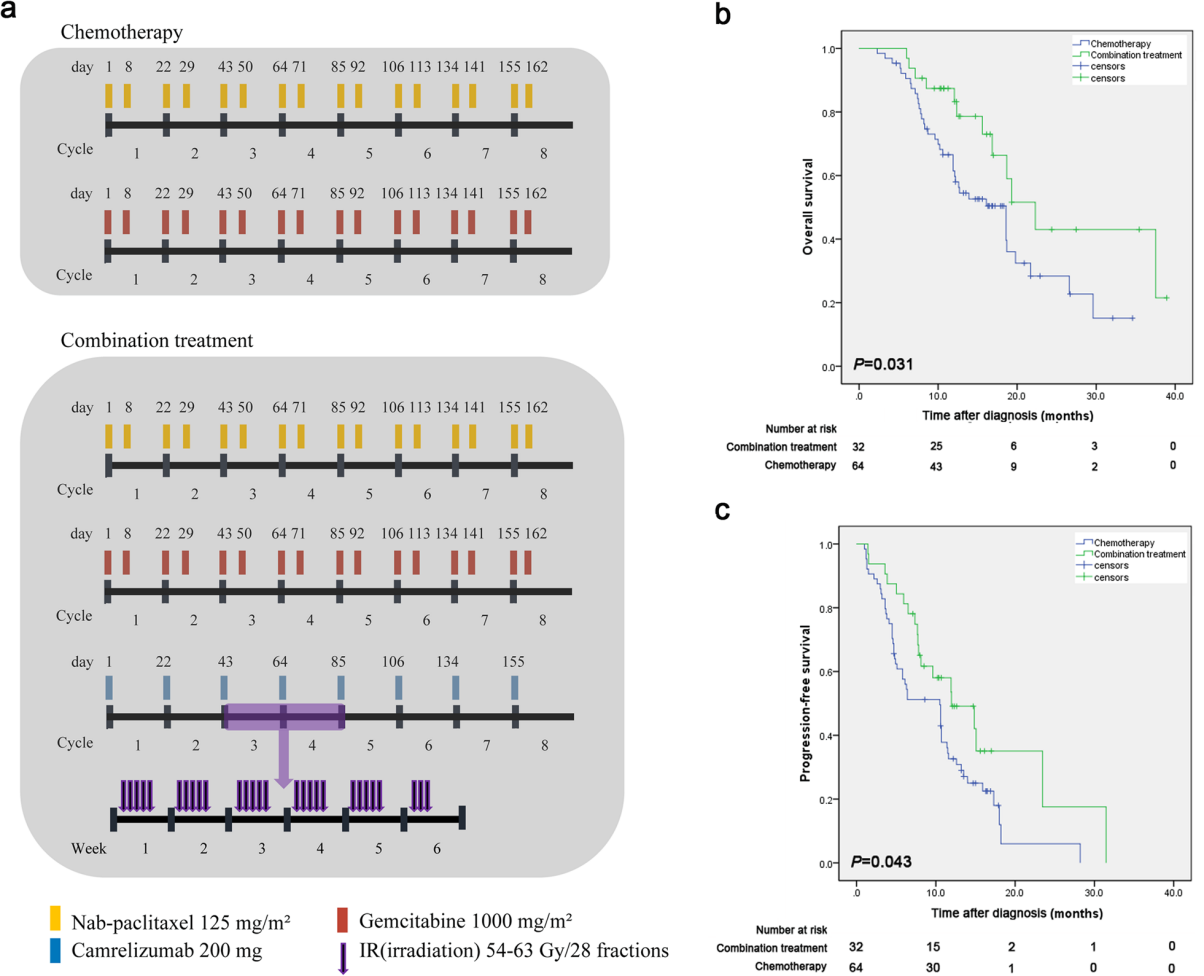
A schematic diagram of two treatment modalities and survival analyses of the patients with locally advanced pancreatic carcinoma who underwent the combination treatment or chemotherapy alone. **a** A schematic diagram of the combination treatment and chemotherapy; **b** Kaplan–Meier curves of overall survival for participants with locally advanced pancreatic carcinoma who underwent the combination treatment or chemotherapy alone; **c** Kaplan–Meier curves of progression-free survival for participants with locally advanced pancreatic carcinoma who underwent the combination treatment or chemotherapy alone

The objective response rate (ORR) based on the RECIST1.1 criteria was 28.1% in the combination group and 21.9% in the chemotherapy group (*P* = 0.163); while the disease control rate (DCR) based on the RECIST1.1 criteria was 90.6% in the combination group and 78.1% in the chemotherapy group (*P* = 0.163) (Table 1). The median follow-up time was 16.6 (range 12.1–27.5) months in the combination group and 17.9 (range 15.2–26.7) months in the chemotherapy group. The median overall survival (OS) was 22.3 months (95% confidence interval [CI] 16.6, 28.0) in the combination group and 18.6 months (95% CI 13.3, 23.9) in the chemotherapy group (*P* = 0.031) (Table 1; Fig. 1b). The median PFS was 12.0 months (95% CI 5.8, 18.1) in the combination group and 10.5 months (95% CI 6.3, 14.7) in the chemotherapy group (*P* = 0.043) (Table 1; Fig. 1c). Univariable and multivariable analyses showed that only the treatment allocation was the independent prognostic factors of OS (HR = 0.486; 95% CI 0.248–0.952; *P* = 0.035) and PFS (HR = 0.577; 95% CI 0.336–0.992; *P* = 0.047) (Additional file 3: Table S2). During the follow-up, there was no significant difference in the pattern of treatment failure and post-protocol intervention between two groups (all* P* > 0.05) (Additional file 3: Table S3). The results of subgroup survival analyses are shown in Additional file 1: Results and Additional file 2: Fig. S2.

**Table 1:** Summary of tumor response and survival outcomes according to RECIST 1.1 criteria

Outcomes	Combination group (*n* = 32), n (%)	Chemotherapy group (*n* = 64), n (%)	*P* value
Best tumor response			
Complete response	0 (0%)	0 (0%)	
Partial response	9 (28.1)	14 (21.9)	0.499
Stable disease	20 (62.5)	36 (56.3)	0.558
Progressive disease	3 (9.4)	14 (21.9)	0.163
Objective response rate	9 (28.1)	14 (21.9)	0.499
Disease control rate	29 (90.6)	50 (78.1)	0.163
Median OS (mo)*	22.3 ± 2.9 (16.6–28.0)	18.6 ± 2.7 (13.3–23.9)	0.031
Median PFS (mo)*	12.0 ± 3.1 (5.8–18.1)	10.5 ± 2.2 (6.3–14.7)	0.043

*RECIST* Response Evaluation Criteria in Solid Tumors, *OS* overall survival, *PFS* progression-free survival

*Data in parentheses are the 95% confidence interval

Additional file 3: Table S4 shows grade 3 or 4 adverse events (AEs). No unexpected toxicity was observed, and no treatment-related death occurred. The incidence of severe AEs was not significantly different between two groups (81.3% vs. 79.7%; *P* = 0.856). In the combination group, the most frequent (≥ 10% incidence) AEs that were ≥ grade 3 were leukopenia (12 [37.5%]), fatigue (4 [12.5%]), and anemia (4 [12.5%]) (while in the chemotherapy group were leukopenia (21 [32.8%]) and fatigue (7 [10.9%]). Other results regarding treatment interruption, reduction or delay are reported in Additional file 1: Results.

In summary, for the first time, we showed that nab-paclitaxel plus gemcitabine combined with anti-PD-1 immunotherapy and radiotherapy was effective and safe for LAPC patients, and it warrants further investigation in larger randomized trials.

## Supplementary Information

**Additional file 1.** Supplementary Methods and Results.

**Additional file 2.** Supplementary Figures.

**Additional file 3.** Supplementary Tables.

## Data Availability

All data generated or analyzed during this study are included in this published article and its supplementary information files.

## Abbreviations

LAPC: Locally advanced pancreatic adenocarcinoma
IO: Immunotherapy
PD-1: Programmed cell death-1
OS: Overall survival
AE: Adverse event
ORR: Objective response rate
RECIST: Response Evaluation Criteria in Solid Tumors
DCR: Disease control rate
PFS: Progression-free survival
HR: Hazard ratio
CI: Confidence interval
