# Supplementary material for: Nab-paclitaxel and gemcitabine plus camrelizumab and radiotherapy versus nab-paclitaxel and gemcitabine alone for locally advanced pancreatic adenocarcinoma: a prospective cohort study
Source: J Hematol Oncol. 2023 Mar 20;16:26. doi: 10.1186/s13045-023-01422-8 (PMC10026489; doi:10.1186/s13045-023-01422-8)
Supplement: Supplementary file 1 — Additional file 1. Supplementary Methods and Results. [file 13045_2023_1422_MOESM1_ESM.docx]

**Supplementary Materials and methods**

**Study Design**

This prospective cohort study was conducted at a tertiary medical center. It was registered at the www.clinicaltrials.gov (identifier: NCT04365049), and approved by the Institution’s Ethics Committee (approval number: [2020]247). The study was conformed to the standards of the Declaration of Helsinki and all the participants provided written informed consent before enrollment. The inclusion criteria were as follows: (1) aged ≥ 18 years and ≤ 75 years; (2) histologically or cytologically proven diagnosis of pancreatic adenocarcinoma; (3) treatment-naive locally advanced pancreatic cancer (locally advanced status was determined by our multidisciplinary team based on the National Comprehensive Cancer Network definitions [1]); (4) no distant metastasis as defined by CT or MRI of the chest, abdomen and pelvis; (5) at least 1 measurable lesion based on the Response Evaluation Criteria in Solid Tumors criteria (RECIST) 1.1; (6) an Eastern Cooperative Oncology Group (ECOG) performance status score of 0 to 1; (7) life expectancy of at least 3 months; (8) adequate hematological function (absolute neutrophil count≥ 1500 cell/mm^3^, platelet count≥ 100×10^9^/L, hemoglobin concentration> 90 g/L); (9) adequate liver function (total bilirubin< 1.5 times the upper limit of normal; alanine aminotransferase and aspartate aminotransferase≤ 3 times the upper limit of normal; albumin≥ 30 g/L); and (10) adequate renal function (serum creatinine concentration< 1.5 times the upper limit of the normal range or less; creatinine clearance rate≥ 45 mL/min).

The exclusion criteria were as follows: (1) with any other malignancy within the 5 years before enrolment; (2) with active infections (bacterial, viral, or fungal) requiring systematic treatment, with hepatitis B or C infection, or a history of HIV infection, or receiving immunosuppressive therapy; (3) with peripheral sensory neuropathy at a grade> 1; (4) with a history of allergy or hypersensitivity to the study drugs; (5) pregnant or breast feeding women, reproductive aged women who refused to take adequate contraceptive measures during the study; (6) enrolled in other clinical trials within 30 days prior to our study. All patients were informed of the advantages and disadvantages of the two treatment options, including potential treatment outcomes, treatment-related morbidities and costs, and the final treatment decision was generally made by the patients.

**Treatment Interventions**

***Combining Radiotherapy with Immunotherapy and Chemotherapy***

Figure 1a shows a schematic diagram of the two treatment options. Chemotherapy consisted of eight 21-day cycles of nab-paclitaxel plus gemcitabine (nab-paclitaxel 125 mg/m² by intravenous infusion for approximately 30–45 min, followed by gemcitabine 1000 mg/m² intravenous infusion for approximately 30 min on days 1 and 8). Up to two dose reductions for nab-paclitaxel (to 100 mg/m² and 75 mg/m²) and gemcitabine (to 800 mg/m² and 600 mg/m²) were allowed. Chemotherapy was continued until disease progression, death or unacceptable toxicity during the eight treatment cycles. Anti-PD-1 antibody (camrelizumab, Hengrui Medicine Co., Ltd) 200 mg was administered intravenously for 30 min every three weeks until disease progression, death or unacceptable toxicity. During the chemotherapy-treated period, camrelizumab was administered on day 1 of each 21-day cycle before the infusion of chemotherapy. At treating physician’s discretion, the camrelizumab could be temporarily interrupted due to the toxicity, but dose reduction was not allowed. If one of the pharmacological treatments (chemotherapy or camrelizumab) was withheld, the other treatment could be continued.

Radiotherapy started after two cycles of chemotherapy, and was continued until completion or unacceptable toxicity. If the participant did not develop distant metastasis during the first two cycles of chemotherapy, they should receive radiotherapy even they developed local progression. External beam radiation therapy was performed using an intensity modulated radiation therapy technique. The gross target volume included the gross primary tumor and positive regional lymph nodes as defined by the multiphasic imaging, including 4-dimensional computed tomography (CT) and diagnostic magnetic resonance imaging (MRI). The clinical target volume was generated with a 5-mm expansion on the gross target volume with an additional 5 mm for the planning target volume. A total radiation dose greater than 50 Gy without damaging organ function was the essential requirement in the research protocol. The median dose/fraction was 60 Gy/28 fractions (range, 54-63 Gy/28 fractions). The prescription dose was adjusted based on the proximity to the at-risk organ.

***Chemotherapy Alone***

Nab-paclitaxel and gemcitabine were administered intravenously on day 1 and 8 of each 21-day cycle for eight cycles. Specifically, nab-paclitaxel 125 mg/m² was intravenously infused for approximately 30–45 min, followed by the gemcitabine 1000 mg/m² intravenous infusion for approximately 30 min. Dose modification was permitted depending on the grade of AEs, and the dose of gemcitabine could be reduced to 800 mg/m² or 600 mg/m². For severe toxicity (i.e., grade 3 or higher), chemotherapy was delayed until AEs had been reduced to grade 2 or less. Chemotherapy was continued until disease progression, death or unacceptable toxicity occurred during the eight treatment cycles.

**Study Evaluation**

***Survival Outcome.*** Patients were followed up after every 2 cycles of camrelizumab or chemotherapy during the treatment period, and then every 2-month from the end of treatment, or earlier if clinically indicated. OS was defined as the interval between the date of LAPC diagnosis and the date of death or the last follow-up. Progression-free survival (PFS) was defined as the time from the date of LAPC diagnosis to radiologic tumor progression, death or the last follow-up. The follow-up was censored on 30^th^ July, 2022.

***Tumor Response.*** Evaluation of tumor response by contrast-enhanced dynamic CT or MRI was performed at baseline, before the beginning of radiotherapy, after every 2 cycles of camrelizumab or chemotherapy during the treatment period, and then every 2-month follow-up visit after the last treatment, or earlier if clinically indicated. The RECIST 1.1 was used for treatment response evaluation [2]. Assessment of tumor response was performed on the target lesions by two experienced radiologists (S.T.F, Z.P.P, 20 years’ experience in liver imaging;) independently, who were blinded to the patients’ clinical information. Any inconsistency of assessment results was resolved by consensus. The objective response rate (ORR) was defined as the percentage of participants achieving complete response (CR) or partial response (PR), and the disease control rate (DCR) as the percentage of participants achieving CR, PR, or stable disease (SD). The best overall response during treatment was categorized as the final response. Considering the possibility of pseudoprogression with immunotherapy, when progressive disease (PD) was detected by RECIST 1.1 criteria for patients in the combination group, another 200 mg of camrelizumab would be given and repeated imaging evaluation was performed 4 weeks later to confirm whether true progression occurred. If true progression was noted, the first date of progression was applied.

***Safety.*** The participants were monitored by vital signs, physical examination findings, clinical and laboratory tests, and AEs throughout the whole study period (i.e., during each cycle of camrelizumab or chemotherapy, weekly during radiation therapy, and at each follow-up visit). AE was evaluated using the Common Terminology Criteria for Adverse Events (version 4.0). Grade 3 or 4 AEs were defined as severe AEs. The percentage of drug (chemotherapy or camrelizumab) discontinuation and dose reduction due to AEs and the duration of drug administration was also evaluated.

**Statistical Analysis**

Continuous variables were presented as means ± standard deviation and categorical variables as numbers and percentages. Differences between the combination group and chemotherapy group were compared with the t-test for continuous variables and χ2 test for categorical variables. Survival curves were generated by the Kaplan–Meier method and compared by the log-rank test. The prognostic relevance of potential survival predictors was analyzed by the univariable and multivariable Cox proportional hazard regression models. Subgroups were stratified by sex, age (< 65 years *vs.* ≥ 65 years), ECOG performance status (0 *vs.* 1), CA19-9 concentrations (< 500U/mL *vs.* ≥ 500U/mL), lymph node status (positive *vs.* neagative) and biliary stent (yes or no). Then, the forest plot of subgroup analysis was drawn with estimated hazard ratios (HRs) and 95% confidence interval (CIs). Statistical significance was considered as a two-sided *P* value of less than 0.05. The above statistical analysis was performed with the open-source SPSS 20.0 (SPSS Inc., Chicago, IL) and R version 4.0.2 (Stanford University, CA, USA).

**Supplementary Results**

**Efficacy Analysis**

Subgroup analyses showed that patients with ECOG score of 0 could achieve a significantly longer OS time from the combination treatment (HR= 0.26, 95% CI: 0.08-0.87) (Additional file 2: Fig. S2). Regarding PFS, subgroups of patients aged older than 60 years (HR= 0.42, 95% CI: 0.19-0.94), male patients (HR= 0.51, 95% CI:0.27-0.99), patients with tumor size≤ 5 cm (HR= 0.39, 95% CI: 0.17-0.92), patients with CA19-9 level< 500U/mL (HR= 0.47, 95% CI: 0.22-1.00) and patients without biliary stent (HR= 0.43, 95% CI: 0.20-0.91) could yield significantly longer PFS time from the combination treatment (Additional file 2: Fig. S2).

**Patterns of Treatment Failure and Post-protocol Intervention**

During the follow-up, in the combination group, disease progression appeared in 19 patients, including ten with local failure and nine with distant metastasis; while in the chemotherapy group, disease progression occurred in 51 patients, including 22 with local failure and 29 with distant metastasis. There was no significant difference in the pattern of treatment failure between the two groups (*P*= 0.759, *P*= 0.125). In terms of post- protocol interventions for patients with progression, patients in the combination group achieved a higher conversion rate than patients in the chemotherapy group (2/32, 6.3% *vs.* 0/64, 0.0%, *P*= 0.118). Among these two patients, one had R0 resection and one had R1 resection. On the other hand, there was no significant difference in the non-curative therapies between the combination versus chemotherapy groups (Additional file 3: Table S4). Second-line and subsequent chemotherapy including FOLFIRINOX and S-1 was given to 16 (84.2%) patients in the combination group and 41 (80.4%) patients in the chemotherapy group (*P*= 1.000). Targeted therapy (Parp inhibitor) was given to 1 (5.3%) patient in the combination group and 2 (3.9%) patients in the chemotherapy group (*P*= 1.000). Best supportive care was the other alternative treatment (2, 10.5% *vs.* 5, 9.8%, *P*= 1.000).

**Safety Outcomes**

Twenty-five (78.1%) patients completed all the treatments in the combination group, and 51 (79.7%) patients did in the chemotherapy group (*P*= 0.859). The main reasons for discontinuing the treatments were AEs (4 [12.5%] *vs.* 8 [12.5%], *P*= 1.000) and progressive disease (3 [9.3%] *vs.* 55 [7.8%], *P*= 1.000) in the combination group and chemotherapy group, respectively. The median number of chemotherapy cycles that each patient received overall was 6 (range, 3-8) in the combination group, and 6 (range, 2-8) in the chemotherapy group (*P*= 0.745). For the immunotherapy in the combination group, the median number of cycles that each participant received overall was seven (range, 5-38). For radiotherapy, 90.6% (29/32) of participants finished the full planned radiotherapy course, and only three patients did not due to severe AEs for whom a total dose of 18~45Gy was administered.

The median relative dose intensity of nab-paclitaxel was 78.1% (IQR, 66.7-100.0%) in the combination group and 81.2% (IQR, 73.4-100%) in the chemotherapy group (*P*= 0.426); that of gemcitabine was 76.3% (IQR, 64.8-100.0) in the combination group and 77.8% (range, 65.7-100.0) in the chemotherapy group (*P*= 0.214). The median relative dose intensity of Camrelizumab was 87.5% (IQR: 75.0-100.0) in the combination group. The percentage of patients who had at least one dose reduction was 31.3% for nab-paclitaxel, 40.6% for gemcitabine and 0% for Camrelizumab in the combination group; and 32.6% (*P*= 0.526) for nab-paclitaxel and 38.7% (*P*= 0.678) for gemcitabine in the chemotherapy group. The percentage of patients who had at least one dose delay was 28.1% for chemotherapy (nab-paclitaxel plus gemcitabine) and 18.8% for Camrelizumab in the combination group, and 31.2% (*P*= 0.326) for nab-paclitaxel plus gemcitabine in the chemotherapy group. All dose reductions and delays were due to AEs.

**Reference**

Tempero MA, Malafa MP, Al-Hawary M, Behrman SW, Benson AI B, Cardin DB, Chiorean EG, Chung V, Czito B, Chiaro MD, et al. Pancreatic Adenocarcinoma, Version 2.2021, NCCN Clinical Practice Guidelines in Oncology. J Natl Compr Canc Netw 2021; 19(4): 439-57.

Eisenhauer EA, Therasse P, Bogaerts J, Schwartz LH, Sargent D, Ford R, Dancey J, Arbuck S, Gwyther S, Mooney M, et al. New response evaluation criteria in solid tumours: Revised RECIST guideline (version 1.1). Eur J Cancer 2009; 45(2):228–47.
