# Supplementary material for: Nab-paclitaxel and gemcitabine plus camrelizumab and radiotherapy versus nab-paclitaxel and gemcitabine alone for locally advanced pancreatic adenocarcinoma: a prospective cohort study
Source: J Hematol Oncol. 2023 Mar 20;16:26. doi: 10.1186/s13045-023-01422-8 (PMC10026489; doi:10.1186/s13045-023-01422-8)
Supplement: Supplementary file 2 — Additional file 2. Supplementary Figures. [file 13045_2023_1422_MOESM2_ESM.docx]

**Figure S1. Flow chart of this prospective cohort study.** Abbreviations: LAPC= locally advanced pancreatic cancer; combination treatment= nab-paclitaxel and gemcitabine plus camrelizumab and radiotherapy; chemotherapy alone= nab-paclitaxel and gemcitabine alone.


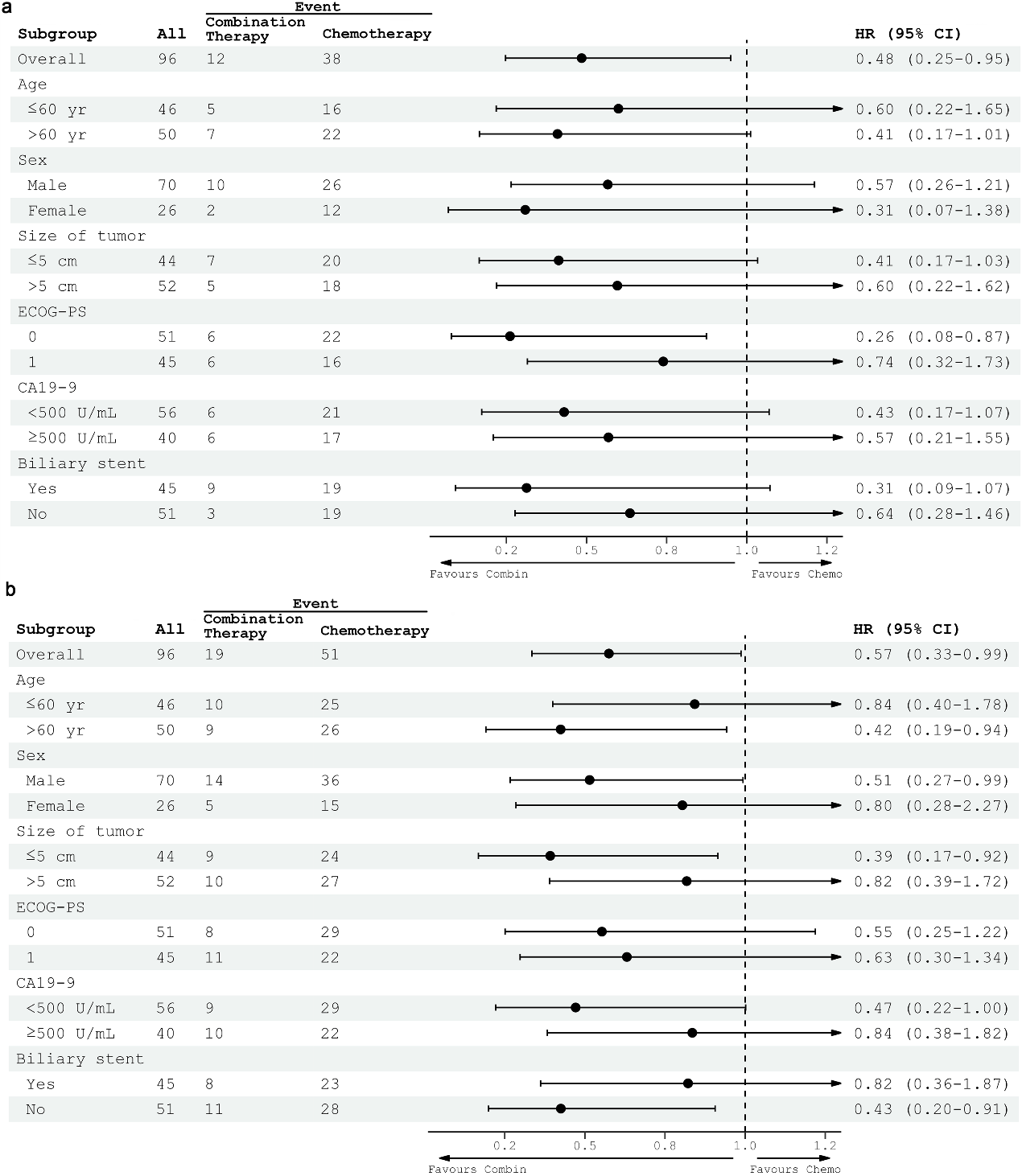


**Figure S2. Subgroup analyses of long-term survivals stratified by pre-defined clinical variables, for participants with locally advanced pancreatic carcinoma who underwent the combination treatment or chemotherapy alone.** (a) Subgroup analysis of overall survival; (b) Subgroup analysis of progression-free survival.
