# Supplementary material for: Nab-paclitaxel and gemcitabine plus camrelizumab and radiotherapy versus nab-paclitaxel and gemcitabine alone for locally advanced pancreatic adenocarcinoma: a prospective cohort study
Source: J Hematol Oncol. 2023 Mar 20;16:26. doi: 10.1186/s13045-023-01422-8 (PMC10026489; doi:10.1186/s13045-023-01422-8)
Supplement: Supplementary file 3 — Additional file 3. Supplementary Tables. [file 13045_2023_1422_MOESM3_ESM.docx]

**Table S1. Demographics and Baseline Clinical Characteristics of Patients with Locally Advanced Pancreatic Cancer in Two Treatment Groups**

| Variables | Combination Group (*n*= 32) | Chemotherapy Group (*n*= 64) | *P* value |
| --- | --- | --- | --- |
| Age (years) |  |  |  |
| Median (range) | 56.5 (38-78) | 54.3 (35-77) | 0.463 |
| ≤ 60 | 15 (46.9) | 31 (48.4) | 0.885 |
| > 60 | 17 (53.1) | 33 (51.6) |  |
| Sex |  |  | 0.731 |
| Male | 24 (75.0) | 50 (78.1) |  |
| Female | 8 (25.0) | 14 (21.9) |  |
| Size of pancreatic tumor, cm |  |  | 0.885 |
| ≤ 5 | 15 (46.9) | 29 (45.3) |  |
| > 5 | 17 (53.1) | 35 (54.7) |  |
| ECOG performance status |  |  | 0.386 |
| 0 | 15 (46.9) | 36 (56.3) |  |
| 1 | 17 (53.1) | 28 (43.7) |  |
| CA19-9 level |  |  | 0.884 |
| < 500 U/mL | 19 (59.4) | 37 (57.8) |  |
| ≥ 500 U/mL | 13 (40.6) | 27 (42.2) |  |
| Lymph node status |  |  | 0.665 |
| Positive | 15 (46.9) | 33 (51.5) |  |
| Negtive | 17 (53.1) | 31 (48.4) |  |
| Biliary stent |  |  | 0.664 |
| Yes | 14 (43.7) | 31 (48.4) |  |
| No | 18 (56.3) | 33 (51.6) |  |

Note. Unless otherwise indicated, data are numbers of patients, and data in parentheses are percentages.

**Table S2. Univariable and Multivariable Cox Regression Analysis of Prognostic Factors of OS and PFS**

| Factor | OS | | | | PFS | | | |
| --- | --- | --- | --- | --- | --- | --- | --- | --- |
|  | Univariable *P* value | Multivariable | | | Univariable *P* value | Multivariable | | |
|  |  | HR | 95% CI | *P* value |  | HR | 95% CI | *P* value |
| Age (≤60/>60 y) | 0.605 |  |  |  | 0.152 |  |  |  |
| Sex (male/ female) | 0.943 |  |  |  | 0.966 |  |  |  |
| Size of tumor (≤ 5/> 5 cm) | 0.435 |  |  |  | 0.442 |  |  |  |
| ECOG (0/1) | 0.966 |  |  |  | 0.668 |  |  |  |
| Baseline CA19-9 (< 500/ ≥500 ng/mL) | 0.337 |  |  |  | 0.323 |  |  |  |
| Lymph node status (Positive/Negative) | 0.145 |  |  |  | 0.701 |  |  |  |
| Biliary stent (Yes/No) | 0.499 |  |  |  | 0.558 |  |  |  |
| Treatment (combination *vs.* chemotherapy) | 0.031 | 0.486 | 0.248-0.952 | 0.035 | 0.043 | 0.577 | 0.336-0.992 | 0.047 |

Abbreviation: OS= overall survival; PFS= progression-free survival; HR= hazard ratio; CI= confidence interval; ECOG= Eastern Cooperative Oncology Group.

**Table S3. Post protocol interventions of the two treatment groups**

| Variables | Combination group  (*n*= 19) | Chemotherapy group  (*n*= 51) | *P* value |
| --- | --- | --- | --- |
| Chemotherapy | 16 (84.2) | 41 (80.4) | 1.000 |
| FOLFIRINOX | 7 (36.8) | 16 (31.4) |  |
| S-1 | 6 (31.6) | 19 (37.3) |  |
| Other | 3 (15.8) | 6 (11.8) |  |
| Target therapy based on gene test (Parp inhibitor) | 1 (5.3) | 2 (3.9) | 1.000 |
| Best supportive treatment | 2 (10.5) | 5 (9.8) | 1.000 |

Note. Unless otherwise indicated, data are numbers of patients, and data in parentheses are percentages.

**Table S4. Treatment-Related Adverse Events (3-4 grade) of the Two Treatment Groups**

|  | Combination Group (*n*= 32) | Chemotherapy Group (*n*= 64) | *P* value |
| --- | --- | --- | --- |
| Any adverse effects | 26 (81.3) | 51 (79.7) | 0.856 |
| General disorders |  |  |  |
| Fatigue | 4 (12.5) | 7 (10.9) | 1.000 |
| Fever | 1 (3.0) | 2 (3.1) | 1.000 |
| Anorexia | 1 (3.1) | 1 (1.6) | 1.000 |
| Weight loss | 2 (6.3) | 3 (4.7) | 1.000 |
| Gastrointestinal disorders |  |  |  |
| Diarrhoea | 2 (6.3) | 1 (1.6) | 0.257 |
| Nausea | 2 (6.3) | 1 (1.6) | 0.257 |
| Vomiting | 1 (3.1) | 1 (1.6) | 1.000 |
| Abdominal pain | 2 (6.3) | 1 (1.6) | 0.257 |
| Hematologic disorders |  |  |  |
| Leukopenia | 12 (37.5) | 21 (32.8) | 0.649 |
| Anaemia | 4 (12.5) | 6 (9.4) | 0.727 |
| Thrombocytopenia | 2 (6.3) | 3 (4.7) | 1.000 |
| Hepatic and renal function abnormity |  |  |  |
| AST elevation | 2 (6.3) | 1 (1.6) | 0.257 |
| ALT elevation | 2 (6.3) | 1 (1.6) | 0.257 |
| Serum bilirubin elevation | 1 (3.1) | 1 (1.6) | 1.000 |
| Serum creatinine elevation | 1 (3.1) | 1 (1.6) | 1.000 |
| Metabolism and nutrition disorders |  |  |  |
| Hypokalaemia | 1 (3.0) | 2 (3.1) | 1.000 |
| Hyponatremia | 1 (3.1) | 3 (4.7) | 1.000 |
| Hypocalcemia | 0 (0) | 1 (1.6) | 1.000 |
| Hypoproteinemia | 2 (6.3) | 5 (7.8) | 1.000 |
| Skin and subcutaneous tissue disorders |  |  |  |
| Alopecia | 1 (3.1) | 1 (1.6) | 1.000 |
| RCCEP | 1 (3.1) | 0 (0) | 0.333 |
| Rash | 1 (3.1) | 0 (0) | 0.333 |
| Pruritus | 1 (3.1) | 1 (1.6) | 1.000 |
| Other systemic diseases |  |  |  |
| Peripheral sensory neuropathy | 1 (3.1) | 1 (1.6) | 1.000 |
| Pneumonitis | 0 (0) | 1 (1.6) | 1.000 |
| Hypothyroidism | 1 (3.1) | 0 (0) | 0.333 |

Note. Unless otherwise indicated, data are numbers of patients, and data in parentheses are percentages.
